# Role of Nod-like Receptors in *Helicobacter pylori* Infection: Insights into Innate Immune Signaling Pathways

**DOI:** 10.3390/microorganisms14020271

**Published:** 2026-01-23

**Authors:** Ah-Ra Jang, Yeong-Jun Kim, In-Su Seo, Wan-Gyu Kim, Sang-Eun Jung, Jong-Hwan Park

**Affiliations:** 1 Nodcure, Inc., 77 Yongbong-ro, Buk-gu, Gwangju 61186, Republic of Korea; 2 Laboratory Animal Medicine, College of Veterinary Medicine and Animal Medical Institute, Chonnam National University, Gwangju 61186, Republic of Koreainsuman1@naver.com (I.-S.S.);

**Keywords:** *Helicobacter pylori*, Nod1 and Nod2, innate immunity, gastric cancer

## Abstract

*Helicobacter pylori* is a prevalent gastric pathogen that establishes chronic infection and contributes to gastritis, peptic ulcer disease, and gastric cancer. Its persistence depends on immune evasion strategies that promote sustained low-grade inflammation in the gastric mucosa. Nucleotide-binding oligomerization domain-like receptors (NLRs) are cytosolic pattern recognition receptors that play key roles in innate immune responses against *H. pylori*. Nod1 and Nod2 detect bacterial peptidoglycan delivered via the type IV secretion system or outer membrane vesicles, activating NF-κB, MAPK, and interferon signaling pathways that regulate inflammatory cytokine production, epithelial barrier function, autophagy, and antimicrobial defense. The NLRP3 inflammasome mediates the maturation of IL-1β and IL-18 primarily in myeloid cells, thereby shaping inflammatory and immunoregulatory responses during infection. In contrast, NLRC4 functions in a context-dependent manner in epithelial cells and is largely dispensable for myeloid IL-1β production. Emerging evidence also implicates noncanonical NLRs, including NLRP6, NLRP9, NLRP12, NLRX1, and NLRC5, in regulating inflammation, epithelial homeostasis, and gastric tumorigenesis. In addition, genetic polymorphisms in NLR genes influence host susceptibility to *H. pylori*-associated diseases. This review highlights the interplay between NLR signaling, bacterial virulence, and host immunity and identifies potential therapeutic targets.

## 1. Introduction

*Helicobacter pylori* (*H. pylori*) is a spiral-shaped, Gram-negative bacterium that chronically colonizes the human gastric mucosa, infecting nearly half of the global population. The organism is typically acquired during childhood and can persist for decades within the harsh gastric environment owing to adaptive mechanisms such as urease-mediated neutralization, flagellar motility, and chemotactic navigation toward the mucus layer. Transmission occurs predominantly through oral–oral or fecal–oral routes, contributing to its widespread distribution and high prevalence in developing regions [1,2].

Although most colonized individuals remain asymptomatic, *H. pylori* persistence elicits a chronic inflammatory response in the gastric mucosa that, in susceptible hosts, can progress to pathological outcomes. Persistent infection can lead to chronic active gastritis, peptic ulcer disease, mucosa-associated lymphoid tissue (MALT) lymphoma, and gastric adenocarcinoma, the latter being one of the leading causes of cancer-related mortality worldwide [3,4]. Importantly, *H. pylori* has evolved sophisticated strategies to evade immune clearance while maintaining low-grade inflammation that ensures its persistence. This delicate balance between bacterial survival and controlled immune activation underlies its capacity to establish chronic infection and contributes to the pathogenesis of gastric malignancy [5].

Detection of pathogens by the immune system is mediated through germline-encoded pattern recognition receptors (PRRs) and soluble effector molecules that respond to conserved microbial components or host-derived danger-associated signals. Major classes of PRRs include Toll-like receptors (TLRs), RIG-I-like receptors (RLRs), and nucleotide-binding oligomerization domain (NOD)-like receptors (NLRs), which are activated by conserved microbial ligands [6,7]. TLRs are differentially localized either on the plasma membrane or within endosomal compartments. Plasma membrane TLRs recognize bacterial surface structures, including lipoproteins, lipopolysaccharide (LPS), and flagellin, whereas endosomal TLRs primarily detect bacterial and viral nucleic acid [6]. In contrast, RLRs and NLRs mediate innate immune responses through cytosolic sensing of bacterial and viral components [7]. In this review, we focus on the roles of NLRs in host immune responses *H. pylori* infection, with particular emphasis on their functions and underlying molecular mechanisms.

## 2. Nod-like Receptors in Innate Immunity

### 2.1. Nod1 and Nod2

**Figure 1:**
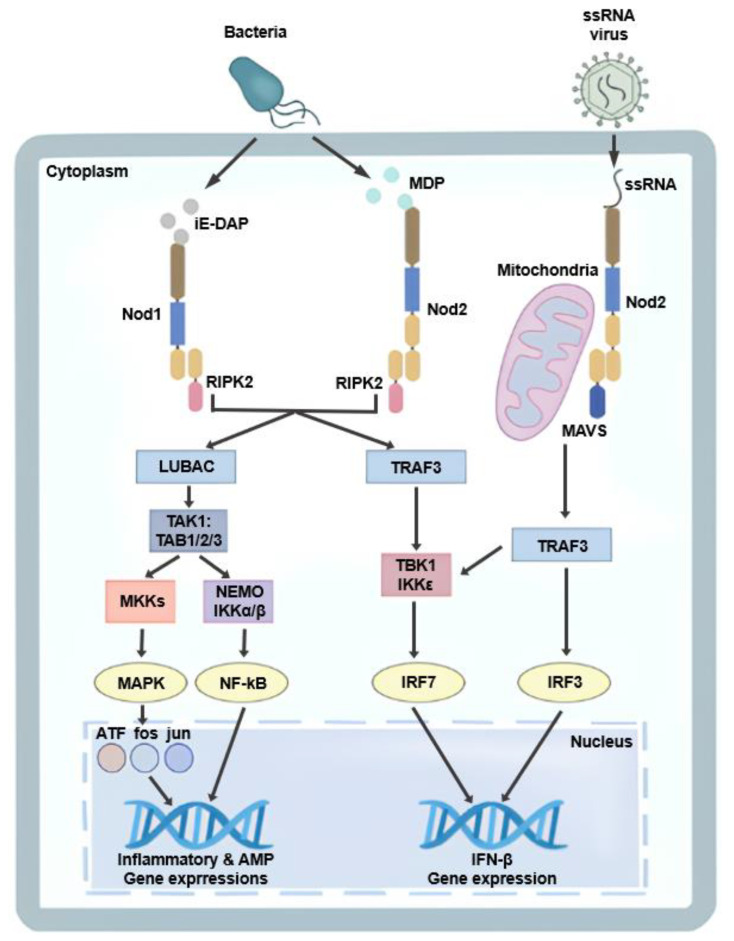
Nod1- and Nod2-mediated signaling pathways. Nod1 and Nod2 detect bacterial peptidoglycan fragments, iE-DAP and MDP, respectively, leading to RIPK2-dependent activation of NF-κB and MAPK signaling pathways and subsequent induction of inflammatory and antimicrobial gene expression. In addition, Nod2 recognizes viral ssRNA and signals through MAVS and TRAF3 to activate TBK1/IKKε, resulting in IRF3/IRF7-mediated type I interferon (IFN-β) production.

Two NLR family members, Nod1 and Nod2, were first identified as mammalian homologues of the apoptotic regulators APAF1 and were subsequently characterized as cytosolic PRRs [8]. Both proteins possess an N-terminal effector domain, a central NOD and C-terminal leucine-rich repeats (LRRs). Nod1 contains a single caspase-recruitment domain (CARD) at its N-terminus, whereas Nod2 contains two CARDs. Both receptors signal through CARD-mediated engagement of receptor interacting serine/threonine kinase (RIP2; also known as RICK), leading to activation nuclear factor kappa-light-chain-enhancer of activated B cells (NF-kB) and mitogen-activated protein kinases (MAPKs) [9,10,11]. These receptors function as intracellular sensors of bacterial peptidoglycan fragments. Nod1 recognizes muropeptide containing γ-D-glutamyl-meso-diaminopimelic acid (iE-DAP), a component of peptidoglycan predominantly found in Gram-negative bacteria and some Gram-positive species. In contrast, Nod2 exhibits broader ligand specificity. In addition to muramyl dipeptide (MDP), Nod2 can sense a wide range of peptidoglycan precursors, including UDP-linked muropeptides, irrespective of the presence of meso-DAP [12]. In addition to bacterial ligands, Nod2 has been reported to recognize virus-derived single-stranded RNA (ssRNA); upon engagement, mitochondrial antiviral signaling (MAVS) and tumor necrosis factor receptor-associated factor 3 (TRAF3) are recruited, resulting in the induction of type I interferon, including IFN-β (Figure 1) [13,14,15].

### 2.2. NLRP3

**Figure 2:**
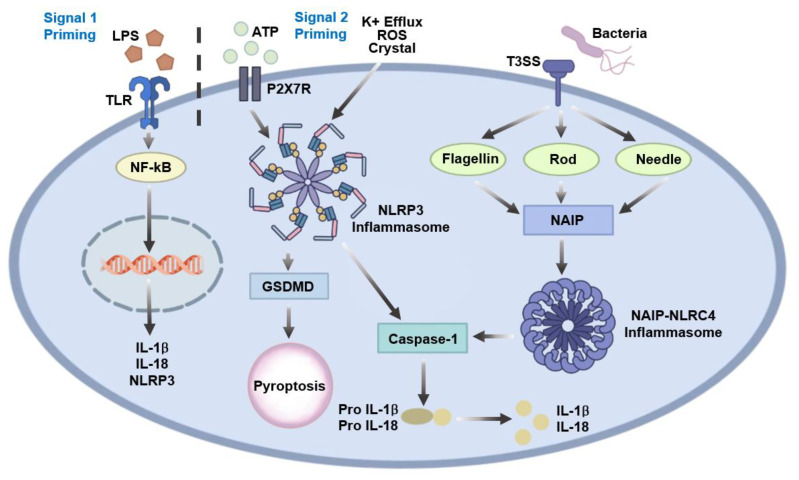
NLRP3 and NLRC4 inflammasome activation pathways. NLRP3 inflammasome activation requires a two-signal model in which priming through TLR- or NLR-mediated NF-κB signaling induces the expression of NLRP3 and pro-IL-1β, followed by activation in response to cellular stress signals such as potassium efflux, mitochondrial ROS, lysosomal damage, or extracellular ATP. Activated NLRP3 assembles with ASC and pro-caspase-1 to promote caspase-1-dependent maturation of IL-1β and IL-18 and induction of pyroptosis via gasdermin D (GSDMD). In contrast, NLRC4 inflammasome activation is directly triggered by NAIP-mediated recognition of bacterial flagellin or T3SS components, leading to caspase-1 activation, pro-inflammatory cytokine maturation, and pyroptotic cell death.

NLRP3 interacts with the adaptor protein apoptosis-associated speck-like protein containing a CARD (ASC) via pyrin domain (PYD)–PYD interactions, which subsequently recruit pro-caspase-1 through CARD–CARD interactions, leading to the assembly of a functional inflammasome complex [16]. Activation of NLRP3 follows a well-established two-signal model. Signal 1 (priming) involves NF-κB-mediated transcriptional upregulation of NLRP3 and pro-IL-1β in response to stimulation of TLRs or NLRs. Signal 2 (activation) is triggered by diverse cellular perturbations, including potassium efflux, lysosomal damage, mitochondrial reactive oxygen species (ROS) generation, or extracellular ATP, which collectively promote inflammasome assembly. Functionally, the NLRP3 inflammasome enables caspase 1-mediated maturation of IL-1β and IL-18 and induces pyroptotic cell death. Through these mechanisms, NLRP3 plays a critical role not only in host defense against pathogens but also in the pathogenesis of a wide range of inflammatory and metabolic diseases, including gout, type 2 diabetes, atherosclerosis, neurodegeneration, and cryopyrin-associated periodic syndromes (Figure 2) [17,18,19].

### 2.3. NLRC4

NLRC4 activation is primarily driven by the intracellular detection of bacterial components, such as flagellin and the type III secretion system (T3SS). This detection is mediated by NLR family apoptosis inhibitory proteins (NAIPs), which serve as upstream sensors that directly bind these microbial ligands. Ligand-bound NAIPs subsequently oligomerize with NLRC4, initiating inflammasome assembly. Unlike NLRP3, NLRC4 activation does not follow the classical two-signal model; instead, ligand recognition by NAIPs directly triggers NLRC4 inflammation formation. Upon activation, the NLRC4 inflammasome induces caspase-1-dependent cleavage of pro-IL-1β and pro-IL-18 and mediates pyroptotic cell death. NLRC4 plays a critical role in the immune defense against intracellular flagellated bacteria, such as *Salmonella*, *Legionella*, and *Pseudomonas*, and is essential for controlling infection through rapid inflammatory responses (Figure 2) [20,21,22]. While this section summarizes the canonical mechanisms of NAIP-NLRC4 inflammasome activation, the relevance of NLRC4 signaling in *H. pylori* infection—an organism that lacks a T3SS and expresses weakly immunostimulatory flagellin—is addressed separately in Section 3.4.

### 2.4. Other NLR Family

NLRC5 acts as a transcriptional regulator of major histocompatibility complex (MHC) class I genes by forming a transcriptional enhanceosome complex in the nucleus. Although initially implicated in innate immunity and antiviral responses, NLRC5 primarily functions in the regulation of antigen presentation, and its deficiency impairs CD8^+^ T cell-mediated immune responses [23,24,25,26].

NLRP6 is highly expressed in intestinal epithelial cells and contributes to gut homeostasis and microbiota composition. Upon activation, potentially in response to microbial metabolites or endogenous stress signals, NLRP6 can recruit ASC and caspase-1 to form an inflammasome complex, thereby enabling the maturation of IL-18 and conferring protection against colitis. Although its precise ligands remain poorly defined, NLRP6 is thought to integrate metabolic and microbial cues in the gut, regulating mucosal barrier integrity and preventing dysbiosis and inflammation [27,28,29].

NLRP9 is also highly expressed in intestinal epithelial cells, particularly during the neonatal period. It recognizes rotavirus double-stranded RNA, likely via cooperation with RNA helicases such as DExH-Box Helicase 9 (DHX9), and forms an ASC-dependent inflammasome that activates caspase-1 and restricts viral replication. NLRP9 plays a crucial role in antiviral defense in the gut mucosa, especially during early development [30,31].

NLRP12, also known as Monarch-1, is unique in its dual role as both a negative and positive regulator of inflammation. Under resting conditions, NLRP12 suppresses noncanonical NF-κB signaling and attenuates inflammatory gene expression. However, in response to specific stimuli, it can also form an inflammasome complex and contribute to caspase-1 activation and IL-1β processing. Though these mechanisms, NLRP12 limits excessive inflammation, and its dysregulation has been implicated in colitis, infectious diseases, and autoinflammatory disorder [32,33].

NLRX1 modulates MAVS and negatively regulates type I IFN and NF-κB signaling pathways in response to viral RNA. In addition, NLRX1 promotes mitochondrial ROS generation, thereby linking metabolic stress to innate immune signaling. Overall, NLRX1 functions as a context-dependent regulator of inflammation and cell death, particularly in viral infections and metabolic disorders [34,35,36].

## 3. The Role of NLRs in *Helicobacter pylori* Infection

### 3.1. Nod1

Nod1 is a cytosolic pattern recognition receptor that plays a central role in sensing peptidoglycan fragments from Gram-negative bacteria and initiating epithelial immune responses during *H. pylori* infection. Although Nod1 and Nod2 share overlapping ligand recognition and downstream signaling pathways, their functions are not fully redundant. Context-dependent differences in Nod signaling, including regulatory or anti-inflammatory roles attributed to Nod2 in certain settings, suggest that Nod1- and Nod2-mediated responses can diverge depending on cellular and infection contexts.

#### 3.1.1. Gene Polymorphism in Nod1

Genetic polymorphisms in Nod1 have been widely studied as determinants of host susceptibility to *H. pylori*-associated gastrointestinal diseases. Numerous studies have shown that these polymorphisms can influence receptor function, downstream signaling, and inflammatory responses [37,38,39,40,41,42,43]. Among the Nod1 polymorphisms identified to date, the rs2075820 (G796A; E266K) variant is the most extensively investigated. This nonsynonymous substitution, in which glutamic acid residue is replaced by lysine within the NOD/NACHT domain, has the potential to alter the protein conformation, ligand responsiveness, and downstream signaling [42]. Functional studies indicate that this variant may modify Nod1-mediated recognition of bacterial peptidoglycan. Several reports have demonstrated attenuated NF-κB activation and reduced IL-8 production, whereas others have described increased IL-8 or cyclooxygenase-2 (COX-2) expression and exacerbated mucosal inflammation [37,40]. These conflicting observations suggest that the functional consequences of the E266K variant are context dependent. Importantly, reduced Nod1 ligand binding or signaling efficiency does not necessarily equate to a global reduction in innate immune activation, as several Nod1-stimulatory muropeptides are also capable of engaging Nod2, albeit with lower affinity [12,44]. Alterations in Nod1 ligand binding may therefore influence ligand availability and competition between Nod1 and Nod2, thereby reshaping downstream inflammatory responses rather than uniformly dampening them. Epidemiological findings are similarly heterogeneous. While some studies have consistently associated rs2075820 with an increased risk of intestinal-type gastric cancer―particularly among individuals infected with *H. pylori* strain harboring the cag pathogenicity island (cagPAI)―others have been phenotype-specific associations [42]. Notably, the Nod1 E266K polymorphism has been linked to more severe of progressive gastritis and to and increased risk of duodenal ulcer in certain populations [37,38]. Collectively, these observations indicate that Nod1 polymorphisms contribute to distinct clinical outcomes of *H. pylori* infection. Collectively, these findings suggest that Nod1 polymorphisms modulate clinical outcomes of *H. pylori* infection in a complex manner, potentially by altering the balance between Nod1- and Nod2-mediated sensing rather than by exerting a strictly Nod1-specific effect.

#### 3.1.2. Nod1-Mediated Immune Responses

**Figure 3:**
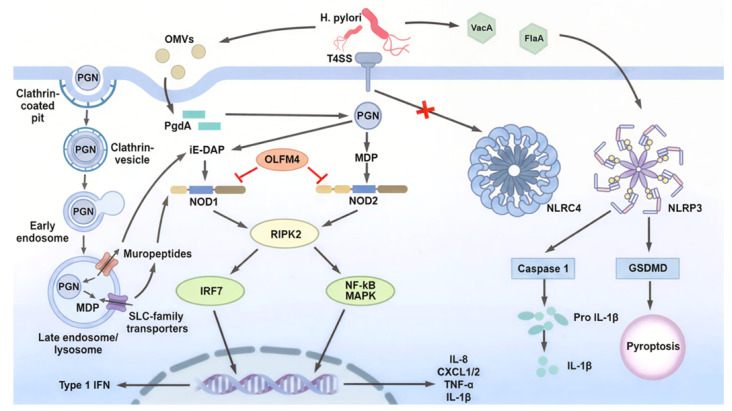
NLR-mediated innate immune signaling during *H. pylori* infection. During *H. pylori* infection, bacterial PGN is delivered into host cells through the T4SS or OMVs, or endocytic uptake pathways, where it is processed into iE-DAP and MDP and sensed by Nod1 and Nod2, respectively. Nod1/2 activation triggers RIPK2-dependent signaling, leading to NF-κB/MAPK and IRF7 activation, which induces the expression of pro-inflammatory cytokines and type I interferons, as well as autophagy through ATG16L1. Host factors such as OLFM4 negatively regulate Nod1 and Nod2 signaling. In contrast, *H. pylori* evades inflammasome activation by limiting direct stimulation of NLRC4, while NLRP3 activation results in caspase-1-dependent maturation of IL-1β and GSDMD-mediated pyroptosis. Together, these pathways illustrate the complex balance between immune activation and immune evasion during *H. pylori* infection.

The role of Nod1 in host immune responses against *H. pylori* infection was first described in 2004 by Viala et al. Nod1 functions as a key sensor in gastric epithelial cells, detecting *H. pylori*-derived components and initiating early innate immune responses [45]. *H. pylori* delivers peptidoglycan (PGN) fragments into the host cytosol primarily through a type IV secretion system (T4SS) encoded by the cagPAI. This system interacts with host β1 integrins to form a translocation pore, enabling the delivery of multiple bacterial components, including CagA, PGN, and genomic DNA [45,46,47,48]. Upon recognition of PGN-derived muropeptides, Nod1 undergoes self-oligomerization and recruits RIPK2 through CARD–CARD interactions. This process induces K63-linked ubiquitination and activating the NF-κB and MAPK pathways, leading the production of proinflammatory cytokines and chemokines, including human IL-8 and its murine homologues KC (CXCL1) and MIP-2 (CXCL2), thereby initiating early inflammatory responses [49,50,51,52]. In a murine model of *H. pylori* infection using strain 251, bacterial loads in the stomachs were significantly higher in Nod1-deficient mice than wild-type (WT) animals at 7 and 30 days post-infection, indicating that Nod1 contributes to early bacterial control in response to cagPAI-positive *H. pylori* strains, although a strictly T4SS-dependent effect was not directly demonstrated [45]. Consistent with a protective role for Nod1 during the early phase of infection, the Strober group further showed that Nod1-deficient mice exhibited impaired bacterial clearance 2 weeks after infection with the TN2GF4 strain, which was associated with defective induction of type I interferon and ISGF3 signaling [53]. In contrast, Asano et al. reported low gastric bacterial loads in Nod1-deficient mice 12 months after infection with *H. pylori* 43504 [54]. These observations suggest that Nod1 is critical for bacterial clearance during the early stages of infection but may exert deleterious effects during chronic infection. Further studies are required to clarify whether these discrepancies reflect differences in infection duration, bacterial strain, or host context. In addition to the T4SS-mediated delivery, *H. pylori* can also deliver Nod1-stimulatory PGN via outer membrane vesicles (OMVs), which are internalized by epithelial cells and contribute to Nod1 activation [55,56,57,58]. Kaparakis et al. demonstrated that Cxcl2 expression was significantly reduced in Nod1-deficient mice compared with WT mice one day after administration of OMVs derived from a cagPAI-positive *H. pylori* strain (252), indicating impaired early innate immune activation. Furthermore, Nod1-deficient mice failed to mount a robust anti-*H. pylori* humoral immune response by day 28, whereas WT mice generated substantial antibody titers [55]. Taken together, these findings suggest that multiple routes of PGN release, including T4SS-mediated delivery, OMV-mediated transport, and passive extracellular release of Nod1- and Nod2- stimulatory muropeptides, shape host responses during *H. pylori* infection, providing early antibacterial defense while also contributing to sustained inflammation and downstream pathological changes (Figure 3). Notably, unlike many Gram-negative bacteria, *H. pylori* lacks a clearly characterized homolog of AmpG, the peptidoglycan recycling transporter, which may facilitate the extracellular accumulation of muropeptides and broaden host exposure to Nod1- and Nod2-stimulatory ligands [59,60,61].

- Inflammatory response

During *H. pylori* infection, Nod1 is activated in gastric epithelial cells, leading to the activation of the NF-κB and MAPK (ERK/p38) pathway, which in turn promote the transcription of proinflammatory cytokines and chemokines, as well as antimicrobial peptides such as β-defensin 2 (BD2) [50,51,52,62]. In contrast, JNK activation is T4SS–integrin-dependent but Nod1-independent and primarily regulates epithelial cell motility rather than inflammatory signaling [63]. These mediators contribute to early neutrophil recruitment, mucosal inflammation, and the maintenance and modulation of epithelial barrier integrity [63,64,65]. Moreover, Nod1–NF-κB signaling influences epithelial differentiation. Chronic *H. pylori* infection induces the intestinal epithelial-specific transcription factor Cdx2, a key driver of intestinal metaplasia [54]. Nod1 activates TRAF3, a negative regulator of NF-κB, which suppresses Cdx2 expression and thereby limits precancerous epithelial remodeling. Accordingly, Nod1-deficient mice exhibit increased Cdx2 expression and enhanced intestinal metaplasia during prolonged *H. pylori* infection, highlighting a protective role of Nod1–NF-κB signaling in maintaining epithelial homeostasis [54].

b. Interferon signaling

Watanabe et al. were the first to demonstrate that Nod1 drives type I interferon production during *H. pylori* infection [66]. Cytosolic detection of *H. pylori*-derived PGN activates the Nod1–RIPK2–TRAF3–TBK1–IRF7 signaling axis, leading to the induction of IFN-β. Secreted IFN-β subsequently engages the IFNAR–JAK–STAT pathway to form ISGF3 complex (STAT1–STAT2–IRF9), which promotes the expression of ISGs such as CXCL10 and amplifies type I IFNs responses. Infection of IFNAR-deficient mice or *Stat1*-knockdown Nod1-deficient mice with a cag-positive *H. pylori* strain (TN2GF4) for 2 weeks resulted in impaired bacterial clearance and reduced CXCL10 expression―phenotypes comparable to those observed in Nod1-deficient mice. These findings indicate that Nod1 enhances epithelial antimicrobial defense and modulates mucosal inflammation during *H. pylori* infection by activating the type I IFN-ISGF3 signaling pathway. [53,66]. In addition to type I IFN responses, Nod1 activation by virulent *H. pylori* strains enhances epithelial sensitivity to IFN-γ. Nod1 stimulation augments IFN-γ-driven STAT1 phosphorylation (Tyr701/Ser727) and IRF1 expression, thereby amplifying the production of downstream chemokines such as IL-8 and CXCL10. Gastric biopsies specimens from patients with severe gastritis or gastric cancer exhibit elevated expression of Nod1, CXCL8, IRF1, and CXCL10, supporting the notion that Nod1–IFN-γ crosstalk contributes to heightened mucosal inflammation and disease severity [67]. Collectively, these findings identify Nod1 as a dual regulator of interferon signaling during *H. pylori* infection, promoting type I IFN-mediated antimicrobial defense while simultaneously enhancing IFN-γ-driven inflammatory responses that may exacerbate mucosal pathology.

c. Autophagy pathways

Upon sensing *H. pylori*-derived PGN, Nod1 and Nod2 contribute to recruitment of ATG16L1 to bacterial contact sites, thereby promoting autophagosome formation as a key epithelial defense mechanism against intracellular bacteria [68]. However, *H. pylori* has evolved strategies to evade this recognition. PGN deacetylation mediated by the enzyme *PgdA* reduces the intrinsic immunostimulatory activity of PGN for both Nod1 and Nod2, thereby attenuating autophagy and facilitating bacterial persistence [51,69]. Consistent with this, in a Mongolian gerbil model, infection with a PdgA-deficient *H. pylori* strain (7.13) resulted in reduced gastric mucosal colonization and markedly attenuated inflammation and tumorigenesis compared with infection by the wild-type strain. Moreover, preactivation of the Nod1 pathway prior to infection suppressed *H. pylori*-induced inflammation and carcinogenesis, supporting a protective role for Nod1 signaling in the gastric mucosa [51]. In contrast, sustained exposure to *H. pylori* lysate activates the Nod1–RIP2–NF-κB/ERK signaling pathway, leading to suppression of the transcription factor FOXO4, a key regulator of multiple autophagy-related genes. This suppression reduces both autophagy and apoptosis, thereby promoting chronic infection. Consistent with this mechanism, gastric epithelial cells from Mongolian gerbils infected with *H. pylori* for 90 weeks exhibited decreased Bcl-2 expression and increased BNIP3 expression, accompanied by marked suppression of apoptosis and autophagy. These alterations were associated with the development of hyperplastic gastric lesions, suggesting a potential progression toward malignancy [70].

### 3.2. Nod2

#### 3.2.1. Gene Polymorphism in Nod2

Nod2 single-nucleotide polymorphisms (SNPs) are regarded as important host factors that can alter the function and expression of this innate immune receptor and thereby contribute to interindividual variation in the magnitude of immune responses to *H. pylori* infection, as well as susceptibility to chronic inflammation-driven diseases [71,72]. The interaction between *H. pylori* infection and Nod2 polymorphisms has been proposed as a determinant of risk for gastric cancer and related gastroduodenal disorders [73]. In particular, Nod2 has been implicated in the pathogenesis of gastric MALT lymphoma. A study reported that the R702W mutation (rs2066844) in the Nod2/CARD15 gene is significantly associated with gastric lymphoma, with carriers of the rare T allele exhibiting approximately a twofold higher risk compared with control (odds ratio [OR] 2.4; 95% confidence interval CI 1.2–4.6, P less than 0.044 [74]. This mutation is linked to structural alterations within the LRR domain of Nod2 suggesting that altered pattern recognition may contribute to the development of lymphoproliferative disease. A meta-analysis further reported that the 3020insC variant rs2066847 is associated with an increased risk of gastric cancer and MALT lymphoma [73]. Notably, Nod2 polymorphisms do not uniformly confer increased risk, and some alleles appear to exert protective effects by reducing the likelihood of disease progression. In a cohort of *H. pylori* positive subjects, the C allele of Nod2 rs2111235 was associated with a lower risk of progression of gastric lesions OR about 0.52, while the G allele of rs7205423 showed a similar protective association OR about 0.56 [41]. These findings contrast with reports indicating that the rs7205423 GC genotype is associated with an increased risk of gastric cancer, indicating that the same locus may be linked to divergent clinical outcomes depending on the specific genotype and clinical context. Overall, these data highlight the complex and often bidirectional clinical impact of Nod2 polymorphisms, which varies according to genotype combinations, disease stage, and the surrounding inflammatory milieu. They underscore the need for context-dependent interpretation and refined genetic analysis when assessing how Nod2 variants influence the risk of *H. pylori* associated gastric diseases [71].

#### 3.2.2. Nod2-Mediated Immune Responses

In the context of *H. pylori* infection, Nod2 has been proposed to function as a cytosolic pattern-recognition receptor that modulates IL-1β-associated innate immune responses, particularly in dendritic cells, where it cooperates with TLR2 to promote pro IL-1β expression and shape downstream inflammatory outcomes [58,75]. However, this role appears to be highly cell-type-dependent, as Nod2 is largely dispensable for IL-1β production in neutrophils during *H. pylori* infection [76]. Importantly, in contrast to Nod1, which has been extensively characterized as a principal sensor of *H. pylori*-derived peptidoglycan in gastric epithelial cells [77,78], the functional relevance of Nod2 in *H. pylori*-driven gastric inflammation and disease progression remains comparatively underexplored. Consistent with this emerging distinction between Nod1 and Nod2 signaling, both receptors are upregulated in gastric tissues during *H. pylori* infection in vivo [74]; however, they participate in immune recognition through mechanistically distinct pathways. Whereas Nod1 activation strongly depends on T4SS-mediated PGN delivery into gastric epithelial cells, Nod2 can be engaged through T4SS-independent routes, most notably via OMVs that transport PGN fragments deep into the gastric mucosa [55,79]. OMV-mediated delivery enables nonphagocytic epithelial cells, which intrinsically express low levels of Nod2, as well as resident myeloid cells in deeper mucosal layers, to internalize bacterial PGN and initiate cytosolic Nod2 signaling. By facilitating access of PGN to anatomical niches beyond the reach of classical phagocyte-dependent pathways, *H. pylori* leverages OMVs to sustain Nod2-driven inflammatory responses, a process increasingly recognized as a central contributor to persistent gastric inflammation and chronic gastrointestinal pathology [80]. In addition, beyond T4SS- and OMV-mediated mechanisms, PGN fragments can also access the host cytosol via endocytic uptake pathways, including clathrin-mediated endocytosis, followed by endosomal processing and transport of muropeptides into the cytosol through SLC-family transporters, providing additional routes for Nod1 and Nod2 activation [81].

Following OMV-mediated delivery of *H. pylori*-derived peptidoglycan into the gastric mucosa, Nod2 functions as a cytosolic signaling hub that couples microbial sensing to inflammatory gene expression. Sustained Nod2 engagement necessitates robust intrinsic feedback control, and accumulating evidence identifies olfactomedin-4 (OLFM4) as a critical negative regulator of Nod signaling during *H. pylori* infection [82]. OLFM4 expression is markedly upregulated during *H. pylori* infection and the protein directly binds both Nod1 and Nod2, acting as an NF-κB-inducible negative regulator that attenuates Nod-dependent inflammatory signaling [82,83]. Consistent with this inhibitory role, loss-of-function studies demonstrate that Olfm4-deficient mice exhibit reduced *H. pylori* colonization accompanied by heightened NF-κB activation and increased expression of IL-1β, IL-5, IL-12p70, and MIP-1α. These findings indicate that OLFM4 suppresses mucosal inflammatory responses and thereby facilitates bacterial persistence [82,83]. Collectively, these observations suggest that *H. pylori* exploits OLFM4-mediated negative feedback to fine-tune Nod2 signaling, allowing sufficient immune activation to sustain chronic infection while avoiding effective bacterial clearance (Figure 3).

### 3.3. IL-1β and NLRP3

IL-1β has emerged as one of the strongest determinants of clinical outcomes following *H. pylori* infection. In 2000, El-Omar and colleagues demonstrated that high-producer IL-1 gene cluster polymorphisms (IL-1B −31T/−511T and IL-1RN*2) markedly increase the likelihood of hypochlorhydria, corpus-predominant atrophic gastritis, and gastric cancer in infected individuals [84]. A subsequent study in 2002 refined this concept by showing that these pro-inflammatory host genotypes confer the greatest cancer risk when combined with virulent bacterial strains such as vacA s1/m1 or cagA^+^ strains, thereby establishing a host–pathogen interaction model in which gastric cancer risk is maximized when a pro-inflammatory host background encounters a highly toxigenic *H. pylori* strain [85]. Mechanistic evidence was later provided in 2008, when parietal-cell-specific IL-1β transgenic mice developed progressive gastritis, oxyntic atrophy, metaplasia, and dysplasia even in the absence of infection, with *Helicobacter* exposure further accelerating these lesions through an IL-1RI–NF-κB–MDSC axis [86]. Together, these findings position IL-1β not merely as an inflammatory marker but as a central pathogenic effector linking *H. pylori* infection to gastric atrophy and carcinogenesis [84,85,86].

IL-1β production during *H. pylori* infection is mediated primarily through inflammasomes assembled by NLR-family sensors. In a large case–control study, several NLRP3 polymorphisms (rs12079994, rs3806265, rs4612666) were associated with increased risk of gastric cancer in *H. pylori*-infected individuals [87]. Interestingly, although *H. pylori* possesses both a functional T4SS and flagellin—features that would typically engage NAIP–NLRC4 inflammasomes—clinical and transcriptional evidence supporting a pathogenic role for NLRC4 in *H. pylori*-associated gastric disease is extremely limited. Unlike NLRP3, for which multiple polymorphisms associate with gastric cancer risk and whose activation clearly drives IL-1β-dependent inflammation, NLRC4 lacks corresponding polymorphism data, and reported changes in its expression during infection are inconsistent across studies.

At the level of innate immune signaling, *H. pylori* activates NLR-dependent inflammasome pathways in a cell-type-specific manner. In dendritic cells, TLR2 and Nod2 provide priming signals that induce NLRP3 and pro-IL-1β expression, whereas the cagPAI—particularly the T4SS adhesin CagL—delivers the activation signal required for ASC and caspase-1 assembly [75]. In murine and human monocytic cells, canonical NLRP3 activation is triggered by potassium efflux, mitochondrial ROS, and lysosomal damage, with both cagPAI components and the pore-forming toxin VacA contributing to caspase-1-dependent IL-1β maturation [75]. Neutrophils employ a related but distinct framework in which IL-1β production is fully dependent on the NLRP3–ASC–caspase-1 axis; priming is driven exclusively by TLR2, and activation requires two neutrophil-specific bacterial cues—CagL-dependent T4SS engagement and FlaA-mediated motility—while *H. pylori* flagellin fails to activate TLR5 or NLRC4, indicating a contact-dependent amplification of inflammasome signaling [76]. Downstream of inflammasome activation, *H. pylori* infection can be accompanied by lytic membrane damage with pyroptotic features in a context-dependent manner. In neutrophils, CagL-dependent T4SS engagement promotes caspase-1 activation and IL-1β maturation, as evidenced by reduced cleavage of caspase-1 and IL-1β in the cagL mutant compared with the isogenic WT strain [76]. Consistent with loss of membrane integrity, infection increased LDH release, which was reduced by oxATP, a P2X7 antagonist [76]. In gastric epithelial cells (AGS), an in vitro infection study detected cleaved caspase-1 and cleaved gasdermin D by immunoblotting and linked their modulation to LDH release [88]. Collectively, these findings support a model in which *H. pylori*-driven inflammasome activation can couple cytokine maturation to lytic membrane injury with pyroptotic features, including LDH release and gasdermin D cleavage, in a cell-type-dependent manner.

Beyond its role in IL-1β maturation, NLRP3 contributes to immunoregulation and the establishment of mucosal tolerance. The urease subunit UreB activates a tolerogenic TLR2–MyD88–NLRP3–caspase-1–IL-18 axis in dendritic cells, promoting Treg differentiation; strains lacking UreB colonize the stomach but fail to induce IL-1β/IL-18 production or Treg responses [89]. Complementarily, NLRP3 is required for the development and recruitment of CD11b^+^ cDC2-like dendritic cells during both steady-state conditions and chronic bacterial infection, independent of ASC, caspase-1, or IL-18. Accordingly, Nlrp3-deficient mice exhibit impaired Treg induction, exaggerated Th1 immunity, and reduced colonization [90]. Together, these findings highlight NLRP3 as a central integrator linking microbial sensing to both inflammatory and tolerogenic immune trajectories in gastric mucosa (Figure 3).

### 3.4. NLRC4/NAIP

Although *H. pylori* is flagellated, it largely avoids NAIP–NLRC4 surveillance. Flagellin-induced phosphorylation of NLRC4 at Ser533 occurs in infected cells, but due to species-specific divergence in the D0 domain, *H. pylori* flagellin fails to engage NAIP5 and therefore cannot initiate full NLRC4 inflammasome assembly [91]. This ligand-level evasion parallels the bacterium’s well-characterized avoidance of TLR5 recognition. Consistent with this mechanism, multiple myeloid cells have shown that NLRC4 is dispensable for *H. pylori*-induced IL-1β production; dendritic cells, monocytes, and neutrophils generate IL-1β exclusively through NLRP3-dependent caspase-1 activation [75,76,92].

In gastric epithelial cells, however, NLRC4 can play a distinct and context-dependent role. One model demonstrated that *H. pylori* induces IL-18—but not IL-1β—through a T4SS-dependent NLRC4–caspase-1 axis that modulates IL-17 responses, β-defensin-1 expression, and bacterial persistence [93]. In contrast, another model found that epithelial IL-18 production and infection outcomes are unaffected in Nlrc4-/-, Nlrp3-/-, or Pycard-/- mice, with low epithelial expression of inflammasome genes and no detectable requirement for canonical inflammasome pathways [79]. Together, these findings indicate that while NLRP3 functions as the dominant inflammasome sensor orchestrating IL-1β production across myeloid lineages, NLRC4 serves as a conditional epithelial modulator whose contribution varies according to bacterial strain, experimental context, and tissue-specific expression (Figure 3).

### 3.5. Other NLRs

Beyond Nod1 and Nod2, several noncanonical NLR family members, including NLRP12, NLRX1, NLRC5, NLRP6, and NLRP9, have been implicated in the regulation of host immune responses during *H. pylori* infection.

NLRP12 functions as a negative regulator of NF-kB and MAPK signaling, and its dysregulation has been linked to chronic inflammatory diseases and elevated cancer risk [94,95]. In *H. pylori* infection models, bacterial exposure reduces NLRP12 expression, likely weakening an intrinsic brake on Nod1 and Nod2-dependent NF-kB activation and thereby favors chronic amplification of innate inflammatory signaling [88]. Cohort-based analyses further indicate that NLRP12 polymorphisms, together with variants in CASP1 and CARD8, are associated with increased gastric cancer risk in *H. pylori*-positive individuals. These findings suggest that combined NLRP12 genotyping and infection status define a susceptibility axis linking dysregulated innate immune control to gastric carcinogenesis [87].

NLRX1 localizes to mitochondria and has been implicated in the inhibition of NF-κB signaling as well as in the regulation of mitochondrial stress, autophagy, and reactive oxygen species (ROS) production [34,96]. *H. pylori*-induced downregulation of NLRX1 in vitro suggests that this pathogen may simultaneously disrupt inflammatory checkpoints and mitochondrial stress responses, thereby promoting ROS-dependent DNA damage and genomic instability [97]. Although derived from limited experimental systems, these findings support a model in which NLRX1 links NF-kB-driven inflammatory amplification to mitochondria-mediated metabolic disruption during the transition from chronic gastritis to a tumor-promoting microenvironment.

Beyond NLRP12 and NLRX1, several additional noncanonical NLR family members exhibit altered expression in the setting of *H. pylori* infection, implying that the broader NLR network participates in bacterial manipulation of host immunity [98]. However, most of the available evidence remains correlative, and the stage-specific contributions of individual NLRs to the gastric cancer cascade are still poorly defined [87].

NLRC5 acts as a transcriptional regulator of major histocompatibility complex class I genes and is essential for cytotoxic T lymphocyte-mediated antigen-specific immune surveillance. Altered NLRC5 expression has been reported in *H. pylori*-infected cells and in gastrointestinal lesions [99,100]. Some studies suggest that upregulated NLRC5 enhances cytotoxic T cell surveillance against infected gastric epithelial cells, whereas others propose that NLRC5 contributes to immune editing and sustained inflammation within the tumor immune microenvironment. Consequently, the net protective versus pathogenic role of NLRC5 in *H. pylori*-associated disease remains unresolved [101].

NLRP9 and NLRP6 have been linked to inflammasome signaling and regulation of the intestinal microbiota in models of dysbiosis-related inflammation and tumorigenesis [102,103]. In gastric tissues, NLRP6 expression is globally reduced in cancer and low NLRP6 levels correlate with *H. pylori* infection, advanced TNM stage, lymph node metastasis, and poor survival [104]. The same study proposed that *H. pylori* suppresses NLRP6 transcription in gastric epithelial cells through the PI3K/AKT/FOXO3 pathway, thereby attenuating the tumor suppressive function of NLRP6 and providing more mechanistic support for a pathogenic link between *H. pylori* and NLRP6 [104]. In contrast, although NLRP9 expression is also modulated by *H. pylori* in vitro, its impact on the stepwise progression of gastric lesions remains speculative and is currently inferred primarily from expression-based correlations. These gaps underscore the need for in vivo *H. pylori* infection models that dissect how NLRP9 and NLRP6 shape stomach-specific inflammasome composition, microbiota alterations, and gastric barrier regulation [87].

## 4. Conclusions

NLRs serve as pivotal sensors and modulators of host immune responses during *H. pylori* infection, integrating signals from bacterial components to orchestrate both protective and pathological outcomes. Nod1 and Nod2 initiate early cytosolic detection of peptidoglycan, activating NF-κB, MAPK, and interferon signaling pathways that enhance epithelial defense, cytokine production, and autophagy. NLRP3 functions as the principal inflammasome sensor in myeloid cells, mediating IL-1β and IL-18 maturation, whereas NLRC4 contributes in a context-dependent manner, particularly in epithelial cells. Noncanonical NLRs, including NLRP6, NLRP9, NLRP12, NLRX1, and NLRC5, further modulate inflammation, epithelial homeostasis, and tumor-promoting processes, illustrating the multifaceted role of the NLR network in gastric mucosal immunity. Host genetic polymorphisms in NLRs and their regulators, such as OLFM4, shape susceptibility to chronic infection, inflammation, and gastric malignancy. Collectively, these findings underscore the complex interplay between NLRs-mediated innate immunity and *H. pylori* pathogenesis and provide insights into potential strategies for therapeutic modulation of gastric inflammation and cancer prevention. From a translational perspective, accumulating evidence suggests that NLR signaling pathways may represent promising targets for immunomodulatory strategies in *H. pylori*-associated diseases. Modulation of the Nod1/2–RIPK2 axis has been proposed to enhance epithelial antimicrobial defenses while limiting excessive inflammation, whereas selective targeting of the inflammasome–IL-1β pathway may help attenuate chronic gastric inflammation and reduce inflammation-driven gastric carcinogenesis. In addition, host genetic variation in NLR-related pathways highlights opportunities for personalized risk stratification and tailored therapeutic approaches. Although direct clinical applications remain limited, these insights provide a conceptual framework for the development of NLR-based interventions in *H. pylori* management.

## Data Availability

No new data created or analyzed in this study. Data sharing is not applicable to this article.

## Abbreviations

The following abbreviations are used in this manuscript:

BD2	β-Defensin 2
cagPAI	Cag pathogenicity island
CARD	Caspase-recruitment domain
iE-DAP	γ-D-glutamyl-meso-diaminopimelic acid
LPS	lipopolysaccharide
LRRs	Leucin-rich repeats
MALT	Mucosa-associated lymphoid tissue
MAPKs	Mitogen-activated protein kinases
MAVS	Mitochondrial antiviral signaling
MDP	Muramyl dipeptide
NF-κB	Nuclear factor kappa-light-chain-enhancer of activated B cells
NLRs	Nod-like receptors
NOD	Nucleotide-binding oligomerization domain
OLFM4	Olfactomedin-4
OMVs	Outer membrane vesicles
PGN	Peptidoglycan
PRR	Pattern recognition receptors
RIPK/RIP2	Receptor interacting serine/threonine kinase
RLRs	RIG-I-like receptors
ROS	Reactive oxygen species
ssRNA	Single-stranded RNA
T3SS	Type III secretion system
T4SS	Type IV secretion system
TLRs	Toll-like receptors
TRAF3	TNF receptor-associated factor 3
